# Combinatory FK506 and Minocycline Treatment Alleviates Prion-Induced Neurodegenerative Events via Caspase-Mediated MAPK-NRF2 Pathway

**DOI:** 10.3390/ijms20051144

**Published:** 2019-03-06

**Authors:** Syed Zahid Ali Shah, Deming Zhao, Giulio Taglialatela, Tariq Hussain, Haodi Dong, Naveed Sabir, Mazhar Hussain Mangi, Wei Wu, Mengyu Lai, Xixi Zhang, Yuhan Duan, Lu Wang, Xiangmei Zhou, Lifeng Yang

**Affiliations:** 1 State Key Laboratory for Agrobiotechnology, National Animal Transmissible Spongiform Encephalopathy Laboratory, Key Laboratory of Animal Epidemiology of the Ministry of Agriculture, College of Veterinary Medicine, China Agricultural University, Beijing 100193, China; zahidvet@cuvas.edu.pk (S.Z.A.S.); zhaodm@cau.edu.cn (D.Z.); drtariq@aup.edu.pk (T.H.); dhd0905@cau.edu.cn (H.D.); naveedsabir@upr.edu.pk (N.S.); drmazharmangi114@gmail.com (M.H.M.); wuwei_971221@126.com (W.W.); macavity813@icloud.com (M.L.); zhangxx16@cau.edu.cn (X.Z.); yhduan@cau.edu.cn (Y.D.); wangl@cau.edu.cn (L.W.); zhouxm@cau.edu.cn (X.Z.); 2 Department of Pathology, Faculty of Veterinary Science, Cholistan University of Veterinary and Animal Sciences (CUVAS), Bahawalpur 63100, Pakistan; 3 Mitchell Center for Neurodegenerative Diseases, Department of Neurology, University of Texas Medical Branch at Galveston, Texas, TX 77555-1044, USA; gtaglial@utmb.edu

**Keywords:** transcription factors, astrogliosis, nuclear factor of activated T-cells (NFAT), phosphorylated mitogen-activated protein kinase (MAPK) p38, nuclear factor kappa-b (NF-kB), phosphorylated cAMP response element-binding protein (pCREB), phosphorylated Bcl2-associated death promoter (pBAD), nuclear factor–erythroid2-related factor-2 (NRF2), heme oxygenase 1 (HO-1)

## Abstract

Transcription factors play a significant role during the symptomatic onset and progression of prion diseases. We previously showed the immunomodulatory and nuclear factor of activated T cells’ (NFAT) suppressive effects of an immunosuppressant, FK506, in the symptomatic stage and an antibiotic, minocycline, in the pre-symptomatic stage of prion infection in hamsters. Here we used for the first time, a combinatory FK506+minocycline treatment to test its transcriptional modulating effects in the symptomatic stage of prion infection. Our results indicate that prolonged treatment with FK506+minocycline was effective in alleviating astrogliosis and neuronal death triggered by misfolded prions. Specifically, the combinatory therapy with FK506+minocycline lowered the expression of the astrocytes activation marker GFAP and of the microglial activation marker IBA-1, subsequently reducing the level of pro-inflammatory cytokines interleukin 1 beta (IL-1β) and tumor necrosis factor alpha (TNF-α), and increasing the levels of anti-inflammatory cytokines IL-10 and IL-27. We further found that FK506+minocycline treatment inhibited mitogen-activated protein kinase (MAPK) p38 phosphorylation, NF-kB nuclear translocation, caspase expression, and enhanced phosphorylated cAMP response element-binding protein (pCREB) and phosphorylated Bcl2-associated death promoter (pBAD) levels to reduce cognitive impairment and apoptosis. Interestingly, FK506+minocycline reduced mitochondrial fragmentation and promoted nuclear factor–erythroid2-related factor-2 (NRF2)-heme oxygenase 1 (HO-1) pathway to enhance survival. Taken together, our results show that a therapeutic cocktail of FK506+minocycline is an attractive candidate for prolonged use in prion diseases and we encourage its further clinical development as a possible treatment for this disease.

## 1. Introduction

Prion diseases are a group of transmissible spongiform encephalopathies (TSEs), which are fatal neurodegenerative disorders affecting both humans and animals [1,2,3]. Currently, the human population is at risk of developing the new form of TSE’s termed variant CJD (vCJD) [4]. Prion diseases have a typical long asymptomatic phase of up to several years [5]. The most common feature of all these diseases is the presence of an abnormal, protease-resistant misfolded isoform of the normal cellular prion protein (PrP^c^), termed PrP^Sc^ [4,6]. PrP^Sc^ is highly pathogenic and neurotoxic due to its β-sheet rich conformation, as compared to predominantly α-helical structure of the normal cellular PrP^c^ [7,8]. The accumulation of PrP^Sc^ leads to severe neuroinflammation and neurodegeneration in the brains of affected individuals [9]. The long presymptomatic phase and lack of diagnostic facilities are great challenges for the scientists working on prion diseases [10].

Transcription factors are specialized proteins and, less often, non-coding RNAs involved in the regulation of gene expression. In humans and animals, genes are usually in an off state and transcription factors serve as switches for turning on of these genes [11]. We previously showed that the important targets for FK506 and minocycline were MAPK- NF-kB-CREB-BAD signaling cascades in prion infected hamsters [3]. Another important transcription factor, NRF2, has been shown to promote antioxidant and anti-inflammatory effects in neurodegenerative diseases [12,13]. NRF2 is a master regulator for controlling cellular oxidative stress and it protects against cellular damage produced by excessive reactive oxygen species and other cellular stressors. NRF2 is repressed by a Kelch-like erythroid cell-derived protein with CNC homology (ECH)-associated protein 1 (Keap1), and Cul3/Rbx1 E3 ubiquitin ligase complex under normal physiological conditions. Under cellular stress conditions, NRF2 is translocated into the nucleus to promote transcription of selected antioxidant enzymes that are key to overcoming potential stress-related cell damages [14].

Tacrolimus (FK506) is a well-characterized macrolide immunosuppressive drug, which is approved for the prevention of allograft rejection in solid organ transplant recipients [15]. Tacrolimus binds to the FK506-binding protein (FKBP) and it inhibits calcineurin (CaN), a calcium and calmodulin-dependent serine threonine protein phosphatase, resulting in the inhibition of NFAT signaling cascades and thus suppression of the T-lymphocytes mediated signal transduction pathway leading to transcription of interleukin 2 (IL2) [16]. The immunomodulatory effects of FK506 on MAPK, NF-kB, and NRF2 pathway has been reported previously in neurodegenerative disorders [3,4,17].

Minocycline, a tetracycline derivative, is an inhibitor of microglial activation that efficiently crosses the blood brain barrier and possesses antimicrobial properties. Minocycline has shown cytoprotective role in nerodegenerative disease models featuring inflammation and cell death [18,19,20,21,22,23]. Microglia, also known as neuroglia, are the brain innate immune macrophage-like cells, which are activated by amyloid β oligomers and fibrils. Microglia are normally found in clusters around the amyloid plaques, and they phagocytize and degrade these plaques as an essential part of a clearance mechanism [21]. The primary target of minocycline is postulated to be T cells, and less NF-kB nuclear translocation has been demonstrated in CD^+^ T cells [24]. Minocycline reduces inflammation via modulating MAPK and NRF2 pathways in neurodegenerative disorders, including prion disease cell models [3,11,25,26].

A novel therapeutic strategy towards achieving the objective of neuroprotection in neurodegenerative disorders should be focused on modulating transcriptional activity of targeted genes, so as to promote neuroprotective transcription factors while suppressing neurotoxic transcription factors in the early symptomatic stage of neurodegeneration. We previously demonstrated that FK506 and minocycline modulated transcription factors in the symptomatic and presymptomatic stage of prion infection, respectively. We have further shown that FK506 was more effective in reducing neurodegeneration as compared to minocycline, whereas minocycline was more effective in reducing neuroinflammation compared to FK506 [3]. In our present study, we investigated whether a cocktail of FK506+minocycline would be effective in the symptomatic stage of prion disease to target neuroinflammation and neurodegeneration simultaneously. Particularly, we explored the immunomodulatory and cytoropective effects of the cocktail of FK506+minocycline at a cellular level on the expression of various cell populations in the brains of prion-infected hamsters to determined their effect on prion-related memory impairments and survival.

## 2. Results

### 2.1. FK506+Minocycline Treatment Prolonged Survival of Prion Infected Hamsters

**Figure 1:**
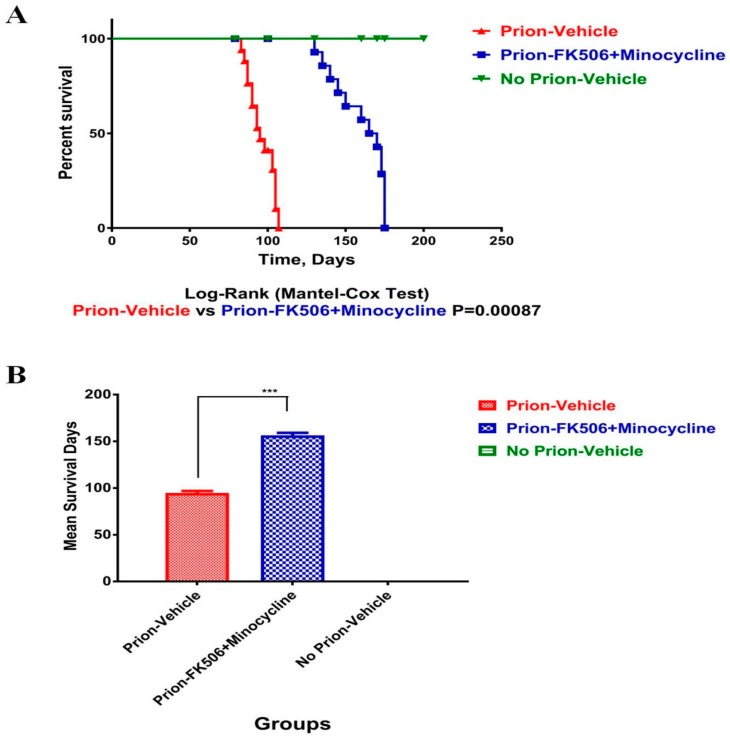
FK506+minocycline treatment prolonged survival of prion infected hamsters (**A**) Statistical representation of survival analysis in prion-vehicle (red color), prion-FK506+minocycline (blue color) and no prion-vehicle group (green color) by Log-rank (Mantel-cox test), number of animals per group were 20. (**B**) Graphical presentation of the infective period in prion-vehicle, prion-FK506+minocycline and no prion-vehicle group. The number of animals per group used for statistical analysis was 20 and the data was analyzed by using one way ANOVA (analysis of variance) test followed by post hoc test tukey’s multiple comparison. (*P* < 0.0001 = ***).

We have previously shown the beneficial effects of early minocycline and late FK506 treatment in the course of prion infection in a hamster model [3]. Here we used a combinatory cocktail of FK506+minocycline in the hamster model at the initial appearance of visible clinical signs on the 79th day post infection and continued the treatment until all animals reached the terminal stage of prion infection. Our results demonstrate that the prion-FK506+minocycline group significantly prolonged survival compared to the prion-vehicle group (Figure 1A,B). The maximum increase in survival time observed in the prion-FK506+minocycline group was 174 days post infection as compared to 107 days post infection survival time in the prion-vehicle group.

### 2.2. FK506+Minocycline Treatment Enhanced Nesting Behavior, Locomotor Function and Novel Object Finding in Prion Infected Hamsters

**Figure 2:**
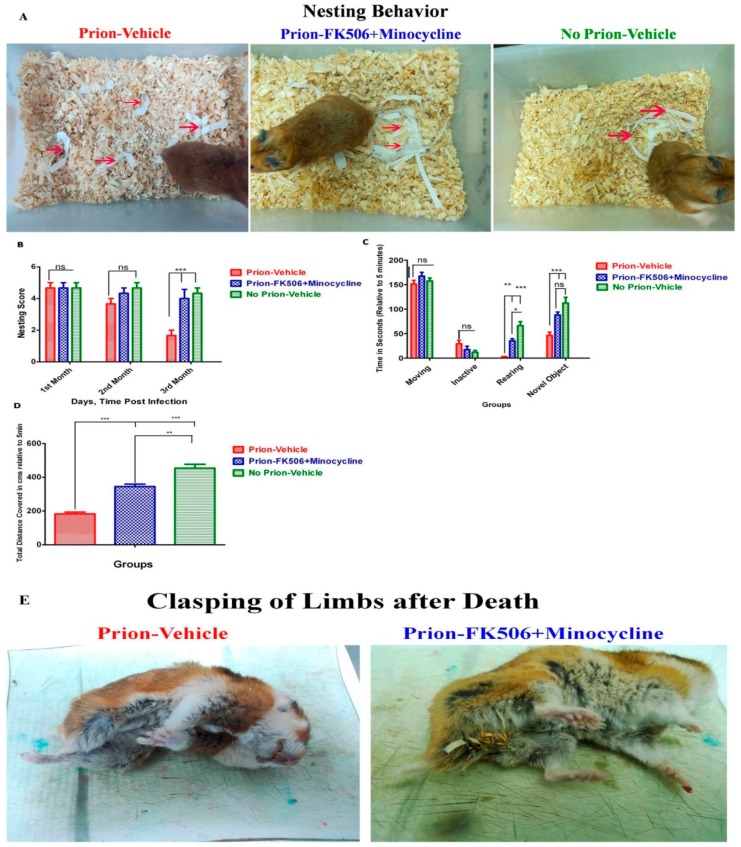
FK506+minocycline treatment enhanced nesting behavior, locomotor function and novel object finding in prion infected hamsters (**A**) Pictures showing the nesting behavior observed in prion infected and non infected animals. Nesting behavior was checked continuously for three months post-infection on a twice-weekly basis. Partially shredded white paper was placed in a top portion of the cage, where the animal does not usually make the nest and the movement of paper to nesting site is shown with red arrows. (**B**) Graphical representation of the nesting score based on the nest quality and movement of the shredded paper from its initial location to nesting site. The graph shows the data obtained from 5 animals each per group. The data was analyzed by using 2 way ANOVA test followed by bonferroni post hoc test. (*P* < 0.001 = *** and *P* > 0.05 = ns). (**C**) Graphical representation of different locomotory activities such as moving activity, inactive period, rearing activity, and novel object exploration duration in different experimental groups relative to 5 min test time, number of animal tested per group were 5. The data was analyzed by using 2 way ANOVA test followed by bonferroni post hoc test. (*P* ≤ 0.01 = **, *P* < 0.001 = *** and *P* > 0.05 = ns). (**D**) Graphical representation of the data showing total distance covered relative to 5 min test time in all experimental groups, number of animal tested per group were 5. The data was analyzed by using one way ANOVA test followed by post hoc test tukey’s multiple comparison. (*P* ≤ 0.01 = ** and *P* < 0.0001 = ***). (**E**) Postmortem appearance of the representative animals from prion infected groups. The clasping of limbs only observed in prion-vehicle group as compared to prion-FK506+minocycvline group.

To evaluate the effect of FK506+minocycline on the nesting behavior of experimentally infected Syrian golden hamsters, we looked at the nest quality of all three experimental groups thrice weekly for twelve weeks until the initial appearance of clinical signs. The nesting score was carried out as previously described in the materials and methods section [27]. Our results illustrate that during the entire initial two month period of the study, there was no significant difference in the nesting behavior among all of the experimental groups (Figure 2A,B). However, during the third month, the nesting performance of the prion-vehicle group was considerably reduced as the disease progressed, whereas the prion-FK506+minocycline group had intact nesting behavior right through the twelve-week test period and beyond (Figure 2A,B). The physical appearance of the representative animals from each group on the 100th day post infection is shown in the supplementary videos (Supplementary Videos S7–S9). The postmortem appearance of animals is very important and our results showed clasping of limbs after death in prion-vehicle group compared to the prion-FK506+minocycline group (Figure 2E). This shows that FK506+minocycline successfully reduced the stress of prion infection.

To examine the locomotory and novel object exploring performance, each animal was separately placed in an open field testing arena and observed for five minutes. Our results explain that there was no significant difference between groups over the first two months post infection trial period. There was no significant dissimilarity in moving and inactive times throughout the third month of the testing period, while a significant difference was noticed in rearing and novel object exploring times in the prion-vehicle and prion-FK506+minocycline group (Figure 2C and Supplementary Videos S10–S12). To further explore locomotory functions, we calculated the total distance covered by each individual animal during the five-minute testing period. We found a significant difference in total distance covered between the prion-vehicle and prion-FK506+minocycline group during the third month testing period (Figure 2D).

### 2.3. FK506+Minocycline Treatment Partially Reduced Calcineurin Activity in Prion Infected Hamsters

**Figure 3:**
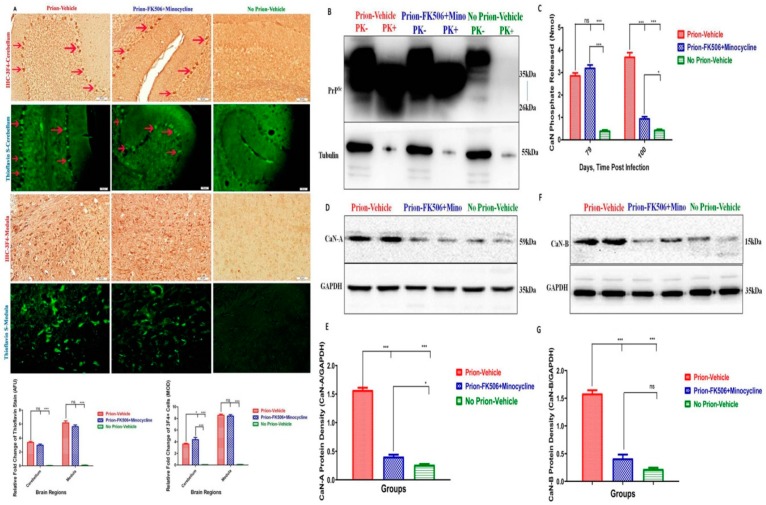
FK506+minocycline treatment partially reduced the calcineurin activity in prion infected hamsters. (**A**) Shows the representative slides for the deposition of PrP^Sc^ in cerebellum and medulla of prion infected and non infected animals stained with 3F4 antibody and thioflavin-S stain (scale bar-50 μm). Prion deposition in cerebellum is shown with red arrows. Bottom of the figure shows the graphical presentation of prion deposition in prion infected and non infected animals. The data represents 5 animals per group and there was no significant difference observed in the cerebellum with thioflavin-S stain, while slight difference was observed the cerebellum after 3F4 stain. The data was analyzed by using two way ANOVA test followed by bonferroni post hoc test. (*P* ≤ 0.05 = *, *P* ≤ 0.01 = **, *P* < 0.001 = ***, *P* > 0.05 = ns) (AFU = arbitrary fluorescence units & MOD = mean optical density). (**B**) Showing representative blots of one animal each per group from prion infected and non infected animals after PK digestion. Total 5 animals from each group were used for western blot analysis. (**C**) Calcineurin activity in response to the accumulation of misfolded proteins, 79th day and 100th day post infection results of prion infected and non infected groups, number of animals tested per group were 3 each time. The data was analyzed by using 2 way ANOVA test followed by bonferroni post hoc test. (*P* ≤ 0.05 = *, *P* < 0.001 = *** and *P* > 0.05 = ns). (**D**) Shows the representative blots of two animals per group for the catalytic unit of calcineurin (calcineurin-A) expression. (**E**) Statistical analysis of calcineurin-A expression in the brain homogenates from different groups, values were normalized using GAPDH as loading control, number of animals used for statistical analysis per group was 5 and the data was analyzed using one way ANOVA test with post hoc test tukey’s multiple comparison. (*P* ≤ 0.05 = * and *P* < 0.0001 = ***). (**F**) Shows the representative blots of two animals per group for the regulatory unit of calcineurin (calcineurin-B) expression. (**G**) Statistical analysis of calcineurin-B expression in the brain homogenates from different groups, values were normalized using GAPDH as loading control, number of animals used for statistical analysis per group was 5 and the data was analyzed using one way ANOVA test followed by post hoc test tukey’s multiple comparison. (*P* < 0.0001 = *** and *P* > 0.05 = ns).

We investigated whether hyperactivated CaN is modulated through the level of misfolded PrP^Sc^ in the brains of the different group of animals. Initially, we carried out immunohistochemistry and theioflavin-S staining of slides from prion-infected and non-infected animals. While the most affected areas of prion aggregation were the cerebellum and medulla, there was no significant difference in prion accumulation as determined by thioflavin-S staining. Similarly, there was a faintly significant difference in prion accumulation between the prion-vehicle group and the prion-FK506+minocycline group in the cerebellum after 3F4 staining, whereas there was no significant difference in prion accumulation among the prion-vehicle group and prion-FK506+minocycline group in the medulla after 3F4 staining (Figure 3A). To further check the level of PrP^Sc^ in the brain homogenates, we quantified PrP^Sc^ levels by western blot. We observed no significant difference in the level of PrP^Sc^ accumulation among all the infected groups of hamsters (Figure 3B). These data suggest that FK506+minocycline acts downstream to PrP^Sc^ accumulation and has no effect on the misfolding nature of the prions. We further checked whether PrP^Sc^ aggregation would lead to CaN hyperactivity. CaN activity was measured at two different time points on the 79th day post infection, just prior to the appearance of initial visible clinical signs, and on the 100th day of infection when the disease progressed. Our results confirmed that CaN activity was significantly higher in the prion-vehicle group compared to the other groups (Figure 3C) and that CaN levels further augmented with the progression of disease. Remarkably, there was no elevation in CaN activity in the prion-FK506+minocycline group after 3 weeks treatment (Figure 3C). We further confirmed through western blot results by detecting the catalytic subunit of calcineurin, CaN A, and the regulatory subunit of calcineurin, CaN B and found consistent results as observed in CaN assay results, demonstrating hyperactivated CaN in the prion-vehicle group compared to other groups (Figure 3D–G).

### 2.4. FK506+Minocycline Treatment Efficiently Reduced Astroglyosis in Prion Infected Hamsters

**Figure 4:**
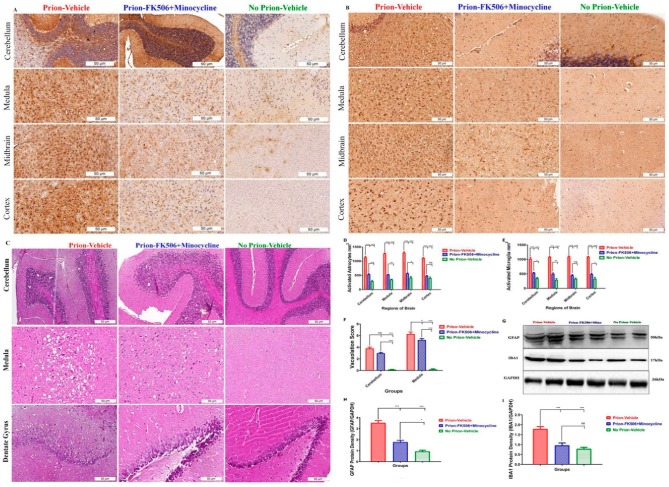
FK506+minocycline treatment efficiently reduced astroglyosis in prion infected hamsters. (**A**) Representative immunohistochemistry pictures of cerebellum, medulla, midbrain, and cortex stained with GFAP antibody for activated astrocytes (scale bar-50 μm). (**B**) Representative immunohistochemistry pictures of cerebellum, medulla, midbrain, and cortex stained with IBA1 antibody for activated microglia (scale bar-50 μm). (**C**) Representative photos of Hematoxylin and Eosin (H&E) stain of cerebellum and medulla for vacuolation profile in prion infected and non infected animals. The bottom three slides show the dentate gyrus neurons in prion infected and non infected animals (scale bar-50 μm). (**D**) The graphical presentation of activated astrocytes in cerebellum, medulla, midbrain and cortex of 5 animals each per group, the data was analyzed by using 2 way ANOVA test followed by bonferroni post hoc test. (*P* ≤ 0.05 = *, *P* ≤ 0.01 = **, *P* < 0.001 = ***, *P* > 0.05 = ns). (**E**) The graphical presentation of activated microglia in cerebellum, medulla, midbrain and cortex of 5 animals per group. The data was analyzed by using 2 way ANOVA test followed bonferroni post hoc test. (*P* ≤ 0.05 = *, *P* ≤ 0.01 = **, *P* < 0.001 = ***, *P* > 0.05 = ns). (**F**) The graphical representation of vacuolation profile in prion infected and non infected groups. Number of animals per group was 5. The data was analyzed by using 2 way ANOVA test followed by bonferroni post hoc test. (*P* ≤ 0.05 = *, *P* < 0.001 = *** and *P* > 0.05 = ns). (**G**) Representative blots of two animals from prion infected and non infected groups for GFAP and IBA1 expression. (**H**) Graphical presentation of GFAP expression in prion infected and non infected animals. Values were normalized using GAPDH as loading control. Number of animals used for statistical analysis was 5 per group. The data was analyzed by using one way ANOVA test followed by post hoc test tukey’s multiple comparison. (*P* ≤ 0.05 = * and *P* < 0.0001 = ***). (**I**) Graphical representation of IBA1 expression in prion infected and non infected animals. Values were normalized using GAPDH as loading control. Number of animals used for statistical analysis was 5 per group. The data was analyzed by using one way ANOVA test followed by post hoc test tukey’s multiple comparison. (*P* < 0.0001 = *** and *P* > 0.05 = ns).

A novel spongiform degeneration, brain vacuolation, and astrogliosis are predominant hallmarks of prion diseases [28]. Therefore, four brain regions including the cerebellum, medulla, midbrain, and cortex were recognized and analyzed for the number of astrocytes and microglia. A highly significant difference in astrocytes activation was observed in the prion-vehicle group as compared to the prion-FK506+minocycline group (Figure 4A,D), suggesting that although the cocktail of FK506+minocycline reduced the number of activated astrocytes in the brains of prion-infected animals, the reduction was incomplete as compared to no prion-vehicle group that had significantly less activated astrocytes (Figure 4A,D). Consistent with our immunohistochemistry analysis, the western blots results for the astrocyte marker GFAP also showed a highly significant difference between prion-vehicle group animals as compared to FK506+minocycline group animals (Figure 4G,H).

Microglial activation plays a vital role in the cellular defense mechanisms against stress stimuli. We observed highly significant activation of microglia in prion-vehicle group animals as compared to prion-FK506+minocycline group animals (Figure 4B,E), suggestive of immunomodulatory effects of the drug treatment on prion mediated microglial activation. Similar results were obtained from western blot analysis of the microglial marker IBA1 (Figure 4G,I).

We found the most prominent vacuolation in the cerebellum and medulla of the brain, where we observed highly significant differences in the vacuolation profile of prion infected and non infected groups (Figure 4C,F). There was a slightly significant difference in the vacuolation profile of the prion-vehicle group as compared to the prion-FK506+minocycline group (Figure 4C,F). Interestingly, we found a large number of vacuoles surrounding the dentate gyrus neurons and loss of the neurons in the prion-vehicle group compared to the prion-FK506+minocycline group (Figure 4C bottom three slides). This shows protection of dentate gyrus neurons afforded by the FK506+minocycline treatment regardless of the number of vacuoles present in this region.

### 2.5. FK506+Minocycline Treatment Rescues Prion Infected Hamsters from Synaptic Dysfunction and Neurodegeneration

**Figure 5:**
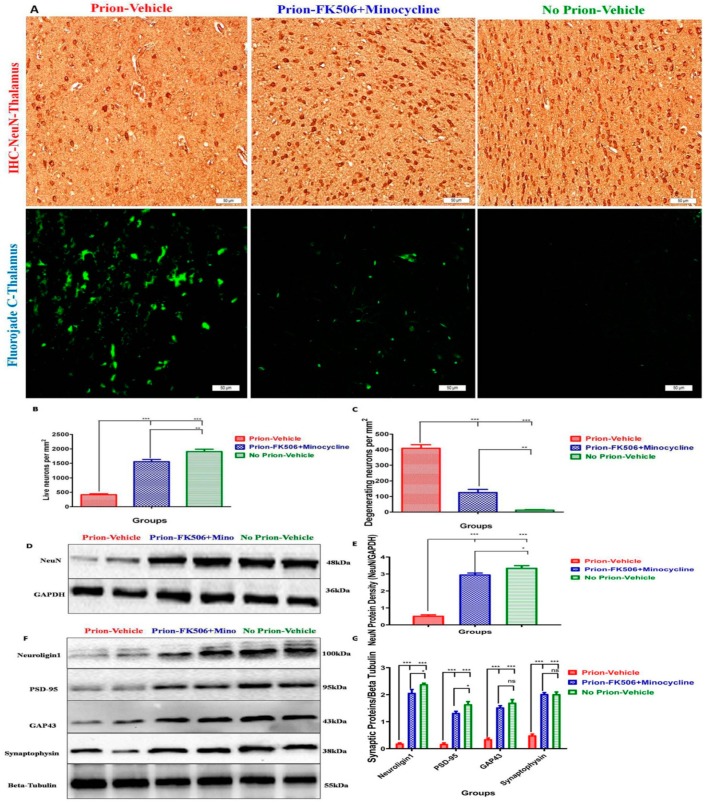
FK506+minocycline treatment rescues prion infected hamsters from synaptic dysfunction and neurodegeneration. (**A**) The top three slides are representative immunohistochemistry pictures of thalamus stained with NeuN antibody to visualize living neurons, while the bottom three slides are representative fluorojade-c stained slides from thalamus for degenerating neurons from prion infected and non infected groups (scale bar-50 μm). (**B**) Graphical presentation of number of neurons per mm^2^ in thalamus of 5 animals per group. The data was analyzed by using one way ANOVA test followed by post hoc test tukey’s multiple comparison. (*P* ≤ 0.01 = ** and *P* < 0.0001 = ***). (**C**) Graphical presentation of the number of degenerating neurons per mm^2^ in thalamus of 5 animals per group. The data was analyzed using one way ANOVA test followed by post hoc test tukey’s multiple comparison. (*P* ≤ 0.01 = ** and *P* < 0.0001 = ***). (**D**) Representative western blot panel of two animals each per group for the expression of NeuN in prion infected and non infected animals. (**E**) Graphical presentation of NeuN expression in prion infected and non infected animals. Values were normalized using GAPDH as loading control. Number of animals used for statistical analysis was 5 per group and the The data was analyzed using one way ANOVA test followed by post hoc test tukey’s multiple comparison. (*P* ≤ 0.05 = * and *P* < 0.0001 = ***). (**F**) Representative western blots of two animals each from every group for protein expression of Neuroligin1, PSD-95, GAP43 and synaptophysin. (**G**) Graphical presentation of the expression levels of Neuroligin1, PSD-95, GAP43 and synaptophysin in the brain homogenates of 5 animals each per group. Values were normalized using beta tubulin as loading control. The data was analyzed by using two way ANOVA test followed by bonferroni post hoc test. (*P* ≤ 0.05 = *, *P* < 0.001 = *** and *P* > 0.05 = ns).

The therapeutic efficacy of any CNS-targeted drug can be evaluated by its effect on neurons. To study the number of living neurons in our experimental circumstances, we fixed 5 brains from every group and immunohistochemical analysis was conducted by using a well-established neuronal marker NeuN [29]. Our results demonstrated a significantly higher number of neurons in the thalamus region in the prion-FK506+minocycline group as compared to prion-vehicle group (Figure 5A Top 3 Slides). Indeed, hamsters in the prion-FK506+minocycline group had almost triple the number of CNS neurons in comparison to prion-vehicle group animals (Figure 5B), suggestive of treatment with FK506+minocycline at the appearance of initial visible signs of prion infection is effective in comparison to prion-vehicle group animals. Though our neuronal analysis demonstrates a significantly higher number of live neurons in the prion-FK506+minocycline group, yet the number of neurons was significantly fewer than the no prion-vehicle group animals, suggesting incomplete protection with FK506+minocycline treatment (Figure 5B). Similar results were obtained from western blot analysis of NeuN in prion infected and non-infected groups (Figure 5D,E).

To evaluate the number of degenerating neurons we carried out Fluoro-jade staining of fixed tissue sections. We found a significantly smaller number of Fluoro-Jade positive cells in the thalamus region of the prion-FK506+minocycline in comparison to prion-vehicle group (Figure 5A bottom three slides and Figure 5C). The effectiveness of the treatment was however modest as the number of Fluoro-Jade positive cells were still significantly high in the prion-FK506+minocycline group in comparison to non-infected vehicle group (Figure 5C).

To assess the effect of our treatment on synaptic dysfunction, we performed western blot analysis for the presynaptic proteins PSD-95, neuroligin1, and the postsynaptic proteins GAP43 and synaptophysin in the brain homogenates of prion infected animals. Our results demonstrate a significant protection of synaptic proteins with FK506+minocycline treatment in comparison to prion-vehicle group hamsters (Figure 5F,G).

### 2.6. FK506+Minocycline Modulates Caspase-Dependent MAPK Pathway in Prion Infected Hamsters

**Figure 6:**
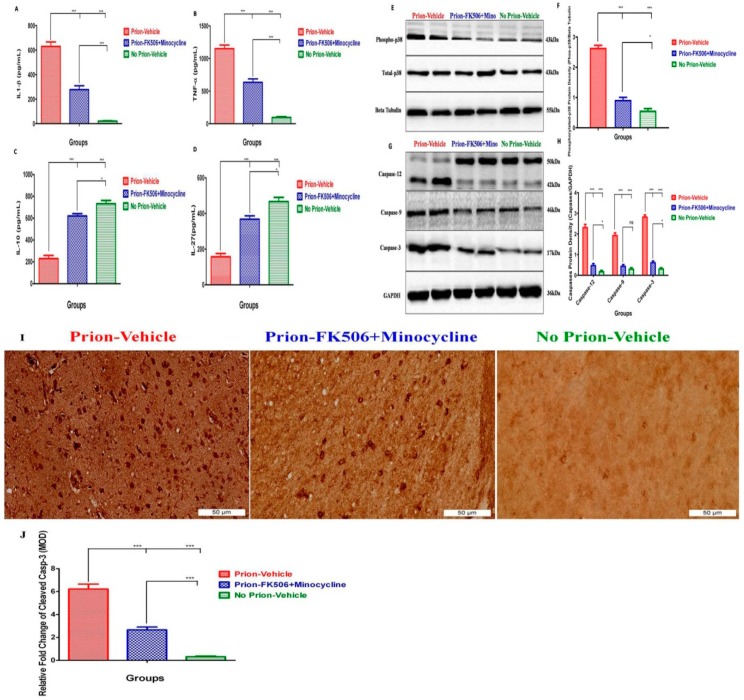
FK506+minocycline modulates caspase-dependent MAPK pathway in prion infected hamsters. (**A**) Showing the level of interleukin 1 beta (IL 1-β) in brain homogenates of 5 animals per group using ELISA technique. The data was analyzed by using one way ANOVA test followed by post hoc test tukey’s multiple comparison. (*P* < 0.0001 = ***). (**B**) Showing the level of tumor necrosis factor alpha (TNF-α) in the brain homogenates of 5 animals per group using ELISA technique. The data was analyzed using one way ANOVA test followed by post hoc test tukey’s multiple comparison. (*P* < 0.0001 = ***). (**C**) Showing the level of tumor necrosis interleukin-10 (IL-10) in the brain homogenates of 5 animals per group using ELISA technique. The data was analyzed using one way ANOVA test followed by post hoc test tukey’s multiple comparison. (*P* ≤ 0.05 = * and *P* < 0.0001 = ***). (**D**) Showing the level of interleukin-27 (IL-27) in the brain homogenates of 5 animals per group using ELISA technique. The data was analyzed using one way ANOVA test followed by post hoc test tukey’s multiple comparison. (*P* ≤ 0.05 = * and *P* < 0.0001 = ***). (**E**) Representative western blots of two animals each from every group for protein expression of MAPK phosphorylated p38 level and total MAPK p38 level. (**F**) Data showing the graphical presentation of the expression level of MAPK phosphorylated p38 and total MAPK p38 level in brain homogenates of 5 animals each per group. Values were normalized using beta tubulin as loading control. The data was analyzed using one way ANOVA test followed by post hoc test tukey’s multiple comparison. (*P* ≤ 0.05 = * and *P* < 0.0001 = ***). (**G**) Representative western blots of two animals each per group for caspase-12, caspase-9 and caspase-3. (**H**) Graphical presentation of the levels of activated caspase-12, caspase-9 and caspase-3 in the brain homogenates of 5 animals each per group. Values were normalized using GAPDH as loading control. The data was analyzed by using 2 way ANOVA test followed by bonferroni post hoc test. (*P* ≤ 0.05 = *, *P* < 0.001 = *** and *P* > 0.05 = ns). (**I**) Representative pictures of immunohistochemical analysis for the expression of cleaved Caspase-3 protein in prion infected and non infected animals (scale bar-50 μm). (**J**) Data showing the protein expression levels of Caspase-3 for apoptosis analysis in different experimental groups based on 5 animals each per group. The data was analyzed by using one way ANOVA test followed by post hoc test tukey’s multiple comparison. (*P* <0.0001 = ***). (MOD= mean optical density).

Microglial activation plays an important role in the release of several proinflammatory and neurotoxic molecules, including major cytokines such as tumor necrosis factor-alpha (TNF-a), interleukin-1 beta (IL-1b), IL-6, and other molecules such as NO, eicosanoids, proteinases, and ROS [30,31]. Similarly, IL-10 and IL-27 are vital for protection against inflammation induced by activated microglia [32,33]. To evaluate the effect of FK506+minocycline treatment on microglia-induced neuroinflammation, we evaluated the levels of proinflammatory cytokines IL-1b and TNF-a, and anti-inflammatory cytokines IL-10 and IL-27 in the brain homogenates of prion infected hamsters. We noticed a significant increase in the levels of IL-1b and TNF-a in prion infected hamsters in comparison to non-infected controls, whereas prion-FK506+minocycline animals had significantly lower levels of IL-1b and TNF-a as compared to prion-vehicle animals (Figure 6A,B). These data propose that FK506+minocycline effectively reduced proinflammatory cytokine levels in the brain of prion infected hamsters in comparison to prion-vehicle animals. In contrast, IL-10 and IL-27 levels were significantly increased in prion-FK506+minocycline group as compared to the prion-vehicle group (Figure 6C,D). Notably, the levels of IL-10 and IL27 in prion-FK506+minocycline group were similar to the no prion-vehicle group, suggesting complete immunoprotection afforded by the cocktail treatment.

Microglial activation controls the release of proinflammatory mediators via activating mitogen-activated protein kinase (MAPK) signaling pathways [34]. To get better insight into the cellular mechanisms mediating the useful effects of FK506+minocycline, we evaluated the level of total and phosphorylated MAPK p38 in the brain homogenates from all prion infected experimental groups. The results showed a significant difference in the level of phosphorylated MAPK p38 between prion-vehicle and prion-FK506+minocycline treated groups (Figure 6E,F), suggestive of the treatment with FK506+minocycline in the symptomatic stage of prion infection and its effectiveness in comparison to prion-vehicle group animals.

Endoplasmic reticulum stress and dysregulated Ca^2+^ leads to caspases activation, ultimately resulting in apoptosis [35,36,37,38]. To examine whether phosphorylation of MAPK p38 trigger caspase induced cell death in response to prion infection and the effects in this manner of the FK506+minocycline, we evaluated the levels of caspase-12, caspase-9, and caspase-3 in the brain homogenates from all investigational groups. We found significantly high levels of caspase-12, caspase-9, and caspase-3 in the prion-vehicle group in comparison to prion-FK506+minocycline group (Figure 6G,H). Similar results were observed when brain slides were stained with cleaved caspase-3 antibody for apoptosis analysis (Figure 6I,J). These data imply that a caspase-mediated phosphorylated MAPK p38 pathway triggers neurodegeneration induced by misfolded prions, and this observable fact was significantly reduced by the cocktail of FK506+minocycline.

### 2.7. FK506+Minocycline Treatment Reduced Mitochondrial Dysfunction in Prion Infected Hamsters

**Figure 7:**
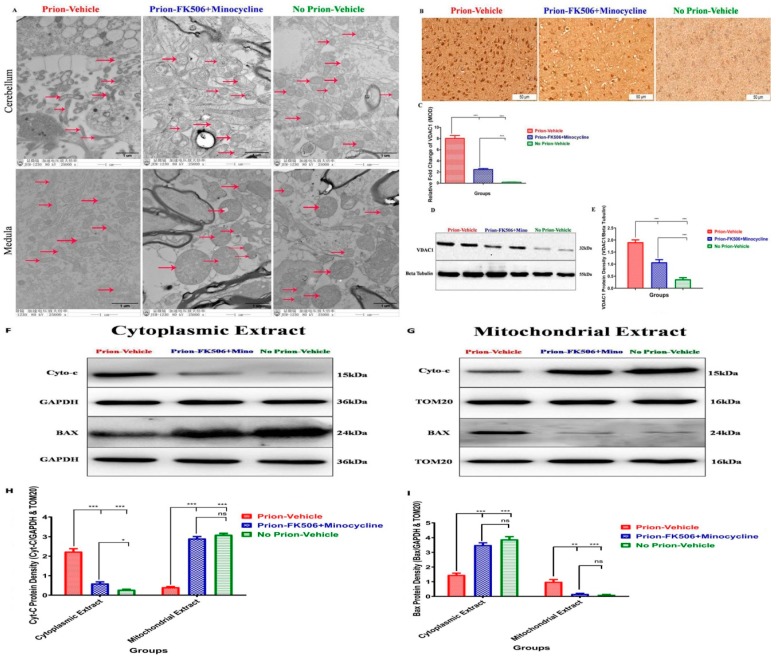
FK506+minocycline treatment reduced mitochondrial dysfunction in prion infected hamsters. (**A**) Representative Transmission Electron microscope images of prion infected and non infected animals. Red arrows represent the mitochondria (80-kV). (**B**) Representative immunohistochemistry slides showing VDAC1 expression in prion infected and non infected animals (scale bar-50 μm). (**C**) The graphical presentation of 5 animals each per group for VDAC1 protein expression in prion infected and non infected animals. The data was analyzed using one way ANOVA test followed by post hoc test tukey’s multiple comparison. (*P* < 0.0001 = ***). (MOD= mean optical density). (**D**) Representative blots of two animals per group for the expression of VDAC1 in prion infected and non infected animals. (**E**) Graphical representation of VDAC1 expression from 5 animals per group, Values were normalized using beta tubulin as loading control. The data was analyzed using one way ANOVA test followed by post hoc test tukey’s multiple comparison. (*P* < 0.0001 = ***). (**F**,**G**) Representative blots of cytoplasmic (left panel) and mitochondrial (right) extracts for the expression of Cytochrom-C and BAX in comparision to GAPDH and TOM20. (**H**,**I**) Graphical presentation of 5 animals each per group for the expression of Cytochrom-C (left side bar graph) and BAX (right side bar graph) in prion infected and non infected animals. Values were normalized using GAPDH and TOM20 as loading controls. The data was analyzed by using two way ANOVA test followed by bonferroni post hoc test. (*P* ≤ 0.05 = *, *P* ≤ 0.01 = **, *P* < 0.001 = *** and *P* > 0.05 = ns).

Endoplasmic reticulum stress leads to mitochondrial dysfunction in many neurodegenerative disorders including prion diseases [36,39]. Our group recently demonstrated that Dynamin-like protein 1 (DLP1) dependent mitochondrial fragmentation occurred in prion infected hamsters and N2a cells [40]. To evaluate the effects of FK506+minocycline treatment on mitochondrial integrity, we conducted electron microscopy analysis of different regions of the brain. Consistent with our previous results, we found severe impairment of mitochondrial integrity in the cerebellum and medulla of prion-vehicle group animals as compared to prion-FK506+minocycline group treated animals (Figure 7A). The crosstalk between mitochondria and endoplasmic reticulum is important in prion diseases and the aggregation of misfolded prion proteins in the endoplasmic reticulum is considered as main cause of apoptotic signals initiation [35,36,41]. To achieve a better understanding of alterations in the mitochondrial dynamics, Voltage-dependent anion-selective channel 1 (VDAC-1) expression was evaluated via immunohistochemistry and western bloting of prion infected and non-infected animals. We found a significantly elevated expression of VDAC1 in the prion-vehicle animals as compared to prion-FK506+minocycline group animals (Figure 7B–E). This suggests that prion infection leads to an opening of the mitochondrial membrane pores and FK506+minocycline kept the integrity of mitochondrial membranes. We further evaluated the levels of cytochrome-C and apoptosis regulator bcl-2-like protein 4 (BAX) in cytoplasm and mitochondria to determine whether an opening of the mitochondrial pores has any effect on movement of cytochrome-C and BAX in prion infection. Our results indicate that FK506+minocycline treatment significantly reduced the translocation of cytochrome-C from mitochondria to cytosol in comparison to prion-vehicle group. In addition, FK506+minocycline significantly reduced BAX relocation from cytosol to mitochondria as compared to the prion-vehicle. The results show that a cocktail of FK506+minocycline efficiently reduced the stress-induced apoptotic signaling in prion infected animals (Figure 7F–I).

### 2.8. FK506+Minocycline Treatment Effectively Leads to Reduced NF-kB p65 and Increased NRF2 Nuclear Translocation

**Figure 8:**
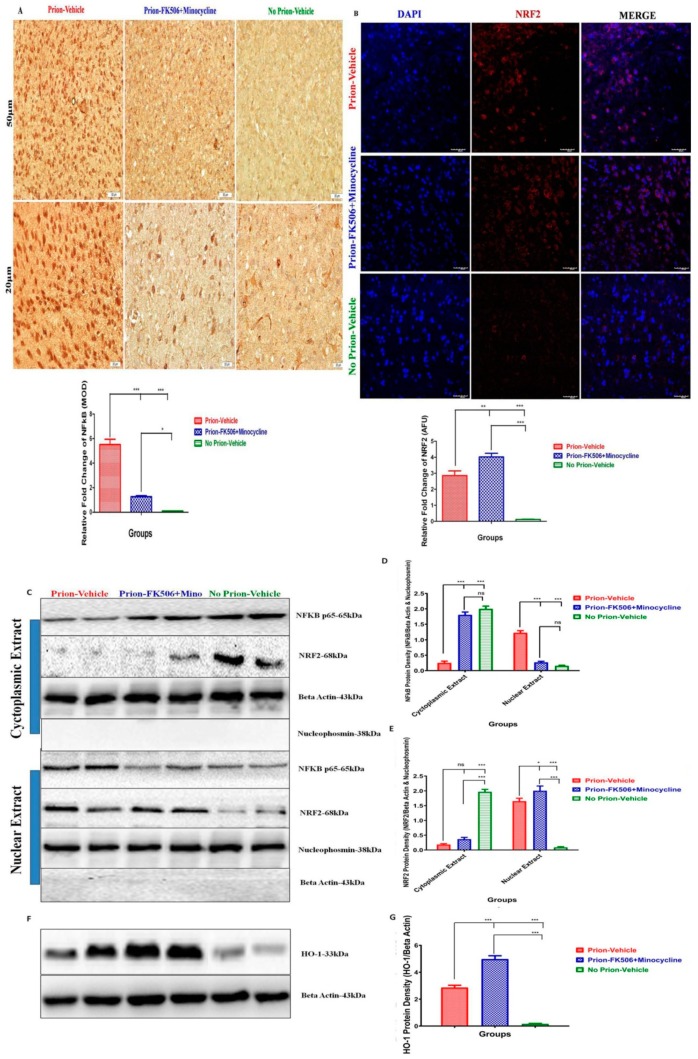
FK506+minocycline treatment effectively leads to reduced NF-kB p65 and increased NRF2 nuclear translocation. (**A**) Representative slides stained with NF-kB p65 adopting immunohistochemistry technique. Top three slides are low power (50 μm) while bottom three slides are high power (20 μm). Graphical presentation at the bottom showing expression of NF-kB p65 in prion infected and non infected groups. Slides from 5 animals each per group were selected for statistical analysis. The data was analyzed using one way ANOVA test followed by post hoc test tukey’s multiple comparison. (*P* ≤ 0.05 = * and *P* < 0.0001 = ***). (MOD= mean optical density). (**B**) Representative pictures of confocal microscopy for nuclear translocation of NRF2 in different groups, First panel stained for nucleus with DAPI (blue), second panel stained for NRF2 (red), third panel is merge of first and second panel. (scale bar-40 μm). The data was analyzed using one way ANOVA test followed by post hoc test tukey’s multiple comparison. (*P* ≤ 0.01 = ** and *P* < 0.001 = ***) (AFU= arbitrary fluorescence units). (**C**) Representative western blot panel of two animals each per group for the protein expression level of NFKB p65 and NRF2 in cytoplasmic extracts (upper panel). (**D**,**E**) The data showing NF-kB p65 and NRF2 protein expression levels in cytoplasm and nucleus of different experimental groups based on 5 animals per group. Values were normalized using beta actin and nucleophosmin as loading controls. The data was analyzed by using 2 way ANOVA test followed by bonferroni post hoc test. (*P* ≤ 0.05 = *, *P* < 0.001 = *** and *P* > 0.05 = ns). (**F**) Representative blots of two animals each per group for the expression of HO-1 in prion infected and non infected animals. (**G**) Graphical presentation of HO-1in the brain homogenates of 5 animals each per group. Values were normalized using beta actin as loading control. The data was analyzed by using one way ANOVA test followed by post hoc test tukey’s multiple comparison. (*P* < 0.0001 = ***).

MAP kinase phosphorylation result in the translocation of nuclear factor kappa-light-chain-enhancer of activated B cells (NF-kB) from cytoplasm to nucleus. Another key transcription factor associated with quick cellular response to oxidative stress is Nuclear Factor–Erythroid2-related factor-2 (NRF2) [14]. To investigate the nuclear translocation of NF-kB and NRF2 we stained the paraffin embedded brain sections with NF-kB p65 and NRF2 antibody. We found significantly high expression of NF-kB p65 in prion-vehicle group animals as compared to prion-FK506+minocycline group animals (Figure 8A). We found augmented NF-kB p65 levels in the nuclear fraction of prion-vehicle group animals brain homogenates as compared to prion-FK506+minocycline group animals in western blot analysis (Figure 8C,D). Furthermore the prion-FK506+minocycline group had nearly twice the levels of NF-kB in the cyctoplasmic portion as compared to the prion-vehicle animals (Figure 8D). These results point out that FK506+minocycline treatment significantly decreased the activation of NF-kB and its subsequent nuclear translocation. These results indicate a useful effect of prion-FK506+minocycline in preventing prion-driven NF-kB nuclear translocation. The nuclear translocation of NRF2 was checked through confocal microscopy, and we found significantly high expression of NRF2 in the prion-FK506+minocycline group in comparison to the prion-vehicle group (Figure 8B). Similarly, in a western blot analysis the levels of NRF2 were significantly increased in the nucleus of the prion-vehicle and prion-FK506+minocycline group as compared to the no prion-vehicle group animals (Figure 8C,E). These results indicate that there is an activation of the NRF2 pathway in response to prion accumulation to overcome the stress signaling. To further elucidate the effect of NRF2 activation on phase 2 detoxifying antioxidant enzymes, we quantified the levels of heme oxygenase-1 (HO-1) in the brain samples of prion infected hamsters. We found a significantly elevated expression of HO-1 in prion-FK506+minocycline treated animals as compared to the prion-vehicle and no prion-vehicle group (Figure 8F,G). This shows that NRF2 activation promotes the antioxidant enzymes to reduce the stress of reactive oxygen species in prion-FK506+minocycline treated animals.

### 2.9. FK506+Minocycline Treatment Increases Cognition and Survival via CREB and BAD Phosphorylation

**Figure 9:**
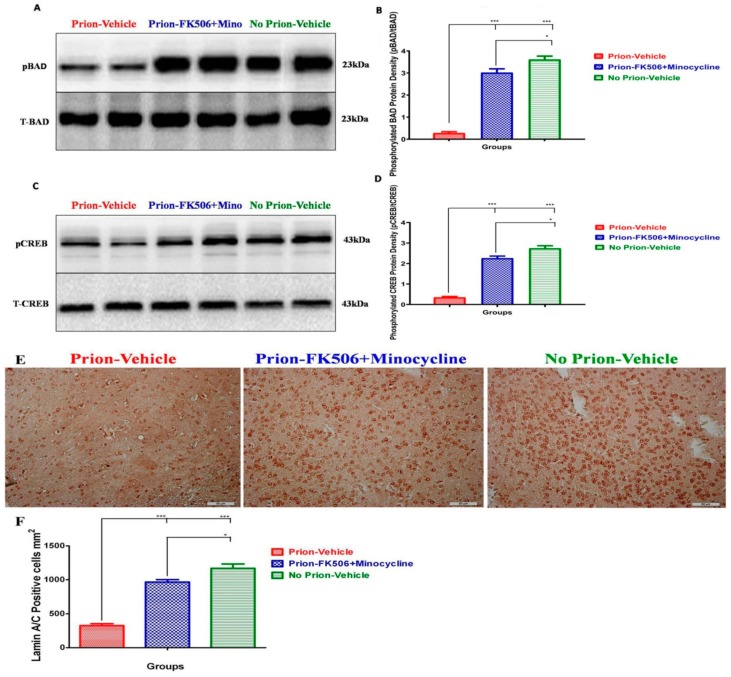
FK506+minocycline treatment increases cognition and survival via CREB and BAD phosphorylation. (**A**) Representative western blot panel of two animals each per group for total BAD and phosphorylated BAD levels. (**B**) Graphical presentation of expression level of phosphorylated BAD in different groups based on 5 animals data per group. Values were normalized using total BAD as loading control. The data was analyzed by using a one way ANOVA test followed by post hoc test tukey’s multiple comparison. (*P* ≤ 0.05 = * and *P* < 0.0001 = ***). (**C**) Representative western blot panel of two animals from each group for total CREB and phosphorylated CREB levels. (**D**) Graph showing protein expression level of phosphorylated CREB in different groups based on 5 animals data per group. Values were normalized using total CREB as loading control. The data was analyzed using a one way ANOVA test followed by post hoc test tukey’s multiple comparison. (*P* ≤ 0.05 = * and *P* < 0.0001 = ***). (**E**) Representative pictures of immunohistochemical analysis for the expression of LMN A/C protein in different groups (scale bar-50 μm). (**F**) Data showing the protein expression levels of LMN A/C for apoptosis analysis in different experimental groups based on 5 animals each per group. The data was analyzed by using a one way ANOVA test followed by post hoc test tukey’s multiple comparison. (*P* ≤ 0.05 = * and *P* < 0.0001 = ***).

To further evaluate the effect of FK506+minocycline on cognition and survival, we checked the levels of Bcl2-associated death promoter (BAD) and cAMP response element-binding (CREB). The western blot analysis of two representative animals from each group is shown in Figure 9; there is a significant differentiation in the levels of pBAD and pCREB in the prion-vehicle group animals as compared to the prion-FK506+minocycline group animals (Figure 9A–D). These results suggest that FK506+minocycline increased survival and enhanced cognition via pBAD and pCREB pathway. Further study of apoptosis was carried out via Lamin A/C immunohistochemical staining in the brain sections. We found a significantly elevated number of Lamin A/C positive cells in the brain of the prion-FK506+minocycline group in comparison to the prion-vehicle group. The prion-vehicle group had very few Lamin A/C positive cells, which were approximately half of the positive cells in the prion-FK506+minocycline group (Figure 9E,F). The animals with no prion infection in the no prion-vehicle group had the highest number of Lamin A/C positive cells, demonstrating the effective, albeit incomplete protection provided by cocktail treatment.

## 3. Discussion

The brain of prion infected individuals is unique with astrogliosis, spongiform degeneration, and neuronal apoptosis [4]. Extensive research efforts have been dedicated to understand the mechanism of molecular events occurring during prion diseases [4,10,42]. The continuous conversion from non-toxic normal cellular PrP^c^ to highly neurotoxic PrP^Sc^ coupled with early neuroinflammation and later neurodegeneration makes it complicated to develop an efficient therapy for prion diseases [43]. Hence there is sufficient consensus that the most efficient therapeutic strategy for prion diseases would be based on a combinatory approach to stop the conversion of normal cellular PrP^c^ into the neurotoxic and misfolded PrP^Sc^ whilst rescuing neurons from initial synaptic dysfunction and neuroinflammation to later neurodegeneration and cell death.

Our treatment approach was based on our group’s previous work where we showed the beneficial effects of minocycline therapy in early presymptomatic and late FK506 treatment in symptomatic stage of prion infection in hamsters [3]. Our hypothesis was that if minocycline therapy effectively rescues neuronal inflammation and FK506 efficiently rescues neuronal degeneration in two different stages of prion diseases, then a combinatory therapy targeting neuroinflammation and neurodegeneration simultaneously in the symptomatic stage might be more effective. FK506 is an immunosuppressant that reduces the elevated activity of the phosphatase calcineurin due to calcium imbalance driven by the accumulation of misfolded prions [3]. The neuroprotective property of FK506 have been established in several neurodegenerative diseases including prion disease [1,4]. Neuroinflammation is vital target for early stage therapeutic interventions in prion diseases. An antibiotic minocycline, a tetracycline derivative, has strong anti-inflammatory, antiapoptotic, and neuroprotective properties [3,44]. Minocycline was selected here not only because of its safety and potential for crossing of blood brain barrier, but also for its documented plaque reducing effects in major neurodegenerative diseases such as Alzheimer’s disease and atherosclerotic models [45,46].

Our results demonstrate significantly useful effects of FK506+minocycline on prion-driven cognitive behavioral abnormalities and survival. Survival analysis shows a significant enhancement in the life of prion infected hamsters after treatment with FK506+minocycline (Figure 1). We found intact nesting behavior for the first two months post infection period in all experimental group animals. By the third month, the nesting behavior impairments of prion-vehicle animals was further exacerbated and FK506+minocycline treatment completely rescued animals from behavioral abnormalities (Figure 2B). We further observed that the nesting behavior was directly proportional to the degree of disease progression, and as the prion-vehicle group animals succumbed to the disease quickly, their nesting behavior diminished quickly in comparison to prion-FK506+minocycline group. Apart from the nesting behavior other locomotory and behavioral tests such as motor activity, rearing activity, novel object finding, exploring time and average distance covered in an open field (OF) test also demonstrated significant differences between the FK506+minocycline and prion-vehicle group, whereas FK506+minocycline treatment counteracted the cognitive behavioral deficits induced by prion infection. These results of an improved cognition and memory in prion-FK506+minocycline group are in agreement with previous reports [20,22,47,48]. The postmortem appearance of animals in prion-vehicle group had clasped limbs in comparison to the prion-FK506+minocycline group animals, where the limbs were wide apart after death (Figure 2E). We further demonstrated that abridged CaN activity in the FK506+minocycline group was not dependent on PrP^Sc^ levels in the prion infected hamsters as shown by the fact that the accumulation of the misfolded aggregated proteins in the brain of prion-vehicle group was parallel to the prion-FK506+minocycline group animals (Figure 2A,B).

The progressive nature of prion diseases involves early neuroinflammatory events leading to later neurodegenerative events. So, controlling the earlier inflammation is regarded as a potential efficient therapeutic approach [21]. Consistent with earlier reports by [4], our data show a significant augmentation in activation of astrocytes in prion-vehicle animals in comparison to the control group (no prion-vehicle) and further found that this phenomenon can be inhibited by the FK506+minocycline treatment. Microglia are an integral part of brain innate immune cells, which are activated in response to the accumulation of misfolded aggregated proteins resulting in the neuroinflammation and subsequent neurodegeneration [21]. Our results show a significant inhibition of microglial activation in prion infected animals receiving FK506+minocycline treatment. These results are in agreement with the previous studies showing anti-inflammatory functions of FK506 and minocycline in unrelated in-vitro and in-vivo models [21,49,50,51,52,53].

The efficacy of a CNS test drug can only be evaluated based on its neuroprotective ability to rescue degenerating neurons and prolong survival. We demonstrated that animals in the prion-FK506+minocycline group had more healthy living neurons and less degenerating neurons in their CNS as compared to the prion-vehicle group. The neuroprotection was moderate and partial as we observed significant differences in the number of healthy neurons in prion-FK506+minocycline group as compared to the no prion-vehicle group. The observed neuroprotective effects of FK506+minocycline are similar to the previous work describing efficient protection of neurons by FK506 after the appearance of initial clinical signs in prion infected mice [1,4].

MAPK pathway plays a crucial role in the neuroinflammatory events leading to axonal degeneration [50,54]. The activation of microglia led to an increased amount of pro-inflammatory cytokines such as IL-1β and TNF-α through MAPK signaling cascades [50,55]. Our results show an elevated level of IL-1β and TNF-α in the prion-vehicle group as compared to the FK506+minocycline treated group. Similarly, anti-inflammatory cytokines IL-10 and IL-27 were checked to further confirm the efficacy of our treatment. We found significantly higher levels of IL-10 and IL-27 in prion-FK506+minocycline treated animals as compared to prion-vehicle treated animals. These results are consistent with the previous work in prion diseases and neuromylitis optica spectrum disorder [3,32,33]. MAP kinase p38 expression was also eminent in the prion-vehicle group in comparison to the prion-FK506+minocycline group. The data propose that FK506+minocycline decrease neuroinflammation via MAPK pathway, which is also in accordance with previous reports [50,51,56,57]. Apoptotic signaling has been linked with several molecules including caspases, and they are well documented in prion and other neurodegenerative disorders [35,36,38,58]. Our data show significantly higher levels of caspase-12, caspase-9, and caspase-3 in prion-vehicle group as compared to the prion-FK506+minocycline treated group. Similar results were obtained for caspase-3 activation to demonstrate neuroprotection with FK506+minocycline treatment in paraffin embedded brain sections. On the one hand, these data corroborate the occurrence of a caspase-mediated apoptotic signaling cascade in prion diseases; they also demonstrate that this neurodegenerative phenomenon is efficiently blocked by FK506+minocycline treatment. These results are in agreement with previous studies in other systems reporting caspases inhibition by FK506 and minocycline [59,60,61,62,63]. Our results using transmission electron microscopy showed that the involvement of mitochondria in prion diseases is consistent with our group’s recently published work, where we reported the involvement of mitochondrial fragmentation in prion diseases [40]. FK506+minocycline efficiently rescued the animals from mitochondrial dysfunction through the inhibition of cytochrome-C from mitochondria and BAD from cytoplasm to mitochondria.

MAPK p38 activation leads to nuclear translocation of transcription factor nuclear factor kappa-B (NF-kB), which subsequently trigger inflammatory mediators [6,57]. Here we demonstrated through western blot analysis of representative cytoplasmic and nuclear extracts obtained from the brain homogenates, that animals in the prion-vehicle group had significantly elevated the amount of NF-kB into the nucleus in comparison to the FK506+minocycline treated animals (Figure 8C). Further study of NF-kB nuclear translocation using immunohistochemistry demonstrated a significantly higher amount of NF-kB protein clustering in or around the nucleus in prion-vehicle group, and to a slighter extent in the prion-FK506+minocycline group animals (Figure 8A), which is constant with previous studies showing abridged nuclear translocation of NF-kB induced by FK506 and minocycline [24,50,64].

The neuroprotective role of activated NRF2 pathway has been described in several neurodegenerative diseases [11,12,65,66,67,68]. Our results using western blot and confocal microscopy confirmed the nuclear translocation of NRF2 in prion infected animals (Figure 8C,E). In addition, the western blot results showed more HO-1 activation after FK506+minocycline treatment as compared to prion-vehicle group (Figure 8F). These results are in accordance with the results of different research groups in other neurodegenerative diseases mentioned above.

When we evaluated the outcome of FK506+minocycline treatment on well known downstream targets of CaN hyperactivation such as pCREB and pBAD, we established that the reduction in pCREB and pBAD induced by prion infection was efficiently prevented by FK506+minocycline treatment (Figure 9B,D). The neuroprotective and prosurvival effect of FK506+minocycline treatment was further established by employing immunohistochemical analysis of apoptotic cells by means of Lamin A/C staining. We found an elevated number of surviving neural cells in animals treated with the FK506+minocycline in comparison to the prion-vehicle group. Further confirmation of neuroprotection was obtained by immunohistochemistry of parafine-embedded brains from infected hamsters. These results are also in agreement with earlier published work presenting protection with minocycline in AD and stroke animal models, and of FK506 in prion disease models [4,69,70].

In conclusion, we have revealed here for the first time that a cocktail of FK506+minocycline, given during the duration of the symptomatic phase of prion infection, efficiently protects from all of the neurodegenerative events and cognitive behavioral deficits that are usually associated with the clinical manifestation of prion disease in hamsters. Taken together, our results recommend that a cocktail of FK506+minocycline should be considered for the clinical development of combinatory therapeutic approach that successfully counteracts the harmful outcomes of prion infections during the clinical phase of the disease.

## 4. Materials and Methods

### 4.1. Ethical Statement

All experimental procedures in the current study were performed and approved according to the stipulated guidelines of Chinese Regulations of Laboratory Animals—The Guidelines for the Care of Laboratory Animals (Ministry of Science and Technology of People’s Republic of China) and Laboratory Animal Requirements of Environment and Housing Facilities (GB 14925–2010, National Laboratory Animal Standardization Technical Committee). The license number of these guidelines was 20110611–01 and our animal study proposal (894843A) was approved on 25th March, 2015 by the Laboratory Animal Ethical Committee of China Agricultural University, Beijing China. Our main aim was to minimize the suffering of the animals, and we kept the number of them studied at a minimum.

### 4.2. Hamster Model of Prion Diseases

The relatively short incubation period (90 days) of prion disease in hamsters and its reproducing ability of many of the clinical, neuropathological, and biochemical aspects of the disease in similarity to humans and other mammals makes them an attractive model for prion diseases [71]. The current study was conducted on a total of 60 female Syrian golden hamsters. All were five-weeks old and housed in separate cages. The animals were given one week time to adjust to the environment and prion infection was given at the age of six weeks. All the animals were divided randomly into three groups and treatment was started when more than half of the animals shown initial symptoms of prion disease on 79th day post infection. The groups were:

- Prion-Vehicle group administered with 0.9% saline started at 79th day post infection
- Prion-FK506+Minocycline group administered with combinatory FK506 and minocycline started at 79th day post infection
- No Prion-Vehicle group administered with 0.9% saline on daily basis started on 79th day post infection.

Group A, B, and C were comprised of 20 animals per group. Animals in group A and B were injected intra-peritoneally (i.p) with 75 µL of 10% brain homogenate prepared from terminally dead hamsters brain infected with 263 K strain of prion in phosphate buffered saline according to previous protocols [72,73]. The initial signs of disease were noticed on 79 ± 7 days post infection. The animals were observed carefully for the appearance of different types of clinical signs and based on these signs animals were divided into five stages 1, normal animal; 2, roughcoat on limbs with erect hairs; 3, extensive roughcoat on limbs with erect hairs, hypersensitivity to noise; 4, hunchback, circling, and some visible motor abnormalities; 5, urogenital lesions, paralysis of back legs and imbalanced posture; 6, terminal stage of the disease in which the animal presented with cachexia and lied in the cage with paddling movement of limbs (Supplementary Videos S1–S6). The time period between the appearance of the initial disease symptoms and death ranged between 10–27 days in animals without any treatment. Treatment administration of the FK506+minocycline was continued until animals died or were sacrificed for the experimental procedures.

### 4.3. Cocktail Preparation and Administration

FK506 (Cat#F-4900) was purchased from LC Laboratories (New Boston Street Woburn, Massachusetts, USA). Purity was ≥99%. Minocycline (M9511) was purchased from Sigma Aldrich (St Louis, MO, USA). Purity was ≥99%. FK506 and minocycline combined stock solution (0.5 mg/mL) was prepared by dissolving the compounds in saline (0.9% NaCl) containing 1.25% PEG40 Castor Oil (Solarbio Life Sciences, Beijing, China, Cat#C9510) and 2% ethanol (Beijing Chemical Works, Beijing, China). For the calculation of best dosage for our study, we conducted a pilot study where we injected different cocktail concentration combinations of FK506 and minocycline (2.5 mg:15 mg, 5 mg:25 mg and 10 mg:40 mg). Animals treated with 5 mg FK506 and 25 mg minocycline in combinatory therapy were found most effective. Animals with 2.5 mg:15 mg did not prolong survival, but with no side effects. On the other hand, 10 mg:40 mg also did not prolong survival, and they had severe weight loss and some other side effects observed in kidney tissues. Animals in group B were injected i.p with 0.12 mg of FK506 (5 mg/kg) and 0.60 mg of minocycline (25 mg/kg) dissolved in 100 mL of the vehicle solution mentioned above [4,22,74,75,76,77]. The stock solution was stored frozen in a light protected bottle.

### 4.4. Reagents

The rabbit polyclonal anti Pan-Calcineurin A antibody (2614) (1:1000) (Cell Signaling Technology, Danvers, MA, USA)). The rabbit polyclonal anti Calcineurin B antibody (AF1348) (0.25 µg/mL) (R&D Systems, Oxford, England). The rabbit polyclonal anti BAD antibody (9292) (1:1000), rabbit polyclonal anti Phosphor-BAD antibody (9291) (1:1000), rabbit monoclonal anti CREB antibody (9197) (1:1000), rabbit monoclonal anti Phospho-CREB antibody (9198) (1:1000), rabbit polyclonal anti p38 MAPK antibody (9212) (1:1000), rabbit polyclonal anti Phospho-p38 MAPK antibody (9211) (1:1000) and rabbit polyclonal anti nucleophosmin (NPM) antibody (3542) (1:1000) were purchased from Cell Signaling Technology. The rabbit polyclonal anti Caspase-12 antibody (55238-1-AP) (1:2000), rabbit polyclonal anti Caspase-9 antibody (10380-1-AP) (1:2000), rabbit polyclonal anti Caspase-3 antibody (19677-1-AP) (1:1000), rabbit polyclonal anti PSD-95 antibody (20665-1-AP) (1:800 WB), rabbit polyclonal anti GAP43 antibody (16971-1-AP) (1:1000 WB), rabbit polyclonal anti synaptophysin antibody (17785-1-AP) (1:800 WB), rabbit polyclonal p65; RELA antibody (10745-1-AP) (1:2000 WB) (1:50 IHC), rabbit polyclonal anti NRF2 antibody (16396-1-AP) (1:1000), rabbit polyclonal anti VDAC1/Porin antibody (55259-1-AP) (1:1000 WB) (1:100 IHC), rabbit polyclonal anti Cytochrome-C antibody (10993-1-AP) (1:1000), rabbit polyclonal anti BAX antibody (50599-2-lg) (1:1000), rabbit polyclonal anti TOM20 antibody (11802-1-AP) (1:1000), rabbit polyclonal anti HO-1 antibody (27282-1-AP) (1:800), rabbit polyclonal anti GFAP antibody (16825-1-AP) (1:50 IHC), rabbit polyclonal anti NeuN antibody (23060-1-AP) (1:50 IHC) and rabbit polyclonal anti IBA1 antibody (10904-1-AP) (1:50 IHC) were purchased from Proteintech Biotechnology (Chicago, IL, USA). The Neuroligin 1 (A-4) (sc-365110) antibody was purchased from Santa Cruz biotechnology, Dallas, TX, USA. The rabbit polyclonal anti LMNA antibody (D120927) (1:50 IHC) from BBI Life Sciences (Sangon Biotechnology, Shanghai, China). The mouse monoclonal anti Prion-3F4 antibody (SIG-39600) (1:1000) (SIGNET-Covance, Emeryville, CA, USA). The rabbit polyclonal Beta Tubulin antibody (10094-1-AP) (1:5000) and mouse monoclonal anti-GAPDH antibody (60004-1-lg) (1:1000) from Proteintech Biotechnology (Chicago, IL, USA). The rabbit polyclonal anti-rat β-actin antibody (Cat No.:AP0060) (1:1000) and goat anti-rabbit IgG (H&L)-HRP secondary antibody (Cat No.:BS13278) (1:5000) were purchased from Bioworld Technology (Nanjing, China). The rabbit anti-goat IgG(H+L) (ZB-2306) (1:5000), Alexa Fluor 594-Conjugated AffiniPure Goat Anti-Rabbit IgG(H+L) (ZF-0516) (1:100) and goat anti-mouse IgG (H&L)-HRP secondary antibody (1:100) were purchased from Beijing ZSGB Biotechnology (Beijing, China). The DAPI dihydrochloride and propidium iodide (PI) were purchased from Beyotime Biotechnology (Wuhan, China). DAB horseradish Peroxidase color Development Kit (P0202) (Beyotime Biotechnology China). Reagents and apparatus used in immunoblotting assays were purchased from Bio-Rad (Richmond, CA, USA). The Colorimetric mouse TNF-α ELISA kit (KE10002) (Proteitech). The IL-10 ELISA kit (KE10008) (Proteintech). The IL-27 ELISA kit (CSB-E08466m) (Cusabio, Houston, TX, USA). The IL-1 beta/IL-1F2 ELISA kit (DY401-05) (R&D Systems).

### 4.5. Assay for CaN Activity

The serine threonine phosphatase activity of CaN was calculated by using the Calcineurin Cellular Activity Assay kit obtained from Calbiochem (Cat#207007) as previously described [4]. CaN cellular activity was first calculated on the 79th day at the appearance of initial clinical signs of the disease, and we again calculated the CaN activity on 100th DPI to see the effect of drugs administration. Briefly, the brain homogenates were prepared in the assay buffer provided in the kit and the residual phosphate was removed by passage through a desalting column. A final concentration of 1 μg/uL of the brain homogenate was evaluated for the enzyme assay in the presence of bovine calmodulin. The obtained reaction mixture was incubated with a final concentration of 150 mM RII peptide (substrate) at 37 °C for 20 min, and the reaction was terminated by the addition of 100 mL malachite green. CaN activity was measured by recording the absorbance at 635 nm wavelength via a micro plate reader (Multiskan FC-51119000) (Thermo Scientific, Waltham, MA, USA).

### 4.6. Animal Behavioral Studies

To examine whether treatment with a cocktail of FK506+minocycline alters clinical signs, we performed Nesting behavior; Open field; and Novel object recognition tests. The Nesting behavior in all groups were observed continuously for 13 weeks and the scoring was done according to previous protocol [27]. Briefly, we placed the partially shredded tissue paper in the cage on opposite side of usual nesting site, and the nesting was scored next day according to the change in location of the shredded paper inside or near the nest. The nest quality was evaluated by using a modified 5-point scale. Tissue was not noticeably touched, moved, or shredded (>90% in same place as originally placed, 1 point); tissue was partially touched, moved, or shredded (50–90% near the nest or inside nest, 2 points); mostly shredded or moved, but not identifiable as a nest (>50% of the paper near the nest or inside the nest, 3 points), an almost intact nest (>90% of the paper near or inside the nest, 4 points); an intact nest (100% of the paper inside the nest, 5 points). The exploratory behavior and locomotory activity in different group of animals were performed through open field (OF) tests as previously described [48], Briefly, the animals tested were individually placed in the 100 × 100 × 40 cm wooden box and they were left to move freely within the box for 5 min. For behavioral tests, a digital video camera (Sony W280-Tokyo, Japan) mounted on top of the open field box was used. The open field box was divided into 15 equal horizontal and vertical lines and all activities during a range of time intervals were recorded. We calculated and analyzed the total distance covered, the moving time, the inactive time, and the rearing activity time in five minutes testing interval. All the tests were performed in a temperature, noise, and light controlled room.

The novel object recognition (NOR) tests were carried out a day after the OF test in the wooden box [78]. The animals were pre-trained to habituate to the box, without the presence of novel object. For testing, the animals were placed individually at the inlet made in the wall of the box and in the presence of four objects of two identical shapes (old objects) for 5 min. The identical shapes objects were placed side by side before test. After that period, the box and objects were cleaned with 50% methanol solution. The animal was later (after 2 h) exposed to the identical objects placed across from each other, while in the center a dummy sheep was placed as a novel object, and the box and objects were cleaned again to continue with the next animal. Recognition index was calculated as the time spent on exploring the new object divided by the time exploring on the old objects.

### 4.7. Detection of PrP^Sc^

The detection and quantification of PrP^Sc^ in the brain homogenates of animals was obtained by a standard assay procedure consisting of the ability of the misfolded prion proteins to resist proteolytic degradation. Samples from the same day slaughtered animals (100th day) and terminally dead animals were incubated in the presence of the proteinase K (75 µg/mL) during one hour with shaking at 37 °C. The digestion of proteins was stopped by adding the electrophoresis sample buffer and protease-resistant misfolded PrP^Sc^ was detected by using western blot as described previously [71].

### 4.8. Western Blotting

Western blot analysis was performed for the same day slaughtered animals (100th DPI) and terminally dead hamsters. We prepared 10% brain homogenate for biochemical tests. The homogenization of frozen brain samples was carried out in RIPA buffer containing a cocktail of protease inhibitors and then sonicated for 15 s, centrifuged at 20,000× *g* for 5 min. The obtained supernatants were collected and then boiled for 10 min after the addition of loading buffer (250 mMTris-HCl pH 6.8, 10% SDS, 0.5% BPB, 50% glycerol, 0.5 M DTT). The protein concentration of each brain sample was calculated before adding loading sample buffer by means of BCA assay (CWBio, Beijing, China) and approximately 30 µg/wel of the protein extract was subjected to western blotting. Samples were separated via SDS-PAGE and the proteins were transferred to PVDF membranes (Immobilon-PSQ, ISEQ00010, 0.2 μm). Blots were blocked by 5% non-fat milk in TBST (25 mMTris base, 137 mM sodium chloride, 2.7 mM potassium chloride and 0.05% Tween-20, pH 7.4) for 1 h at room temperature, incubated with the indicated primary antibody overnight at 4 °C, and the corresponding HRP-labeled secondary antibody for 50 min at 37 °C, and the signal detected using an enhanced chemiluminescence (ECL) detection kit (Bio-Rad, Hercules, CA, USA).

### 4.9. Tissue Preparation and Immunohistochemistry Analysis

Histopathological analysis was carried out to assess the effects of FK506+minocycline treatment on the brain damage. The brains of the animals were taken out surgically under aseptic conditions as quickly as possible after death. The terminally sick animals with signs of scale five were anaesthetized with ketamine:zyline 3:1 (250 µL/100g body weight), then the whole brain was excised and cut sagitally into two equal halves, one half snap frozen in liquid nitrogen for biochemical tests, such as western blot analysis and CaN assay, while the other half was fixed in 10% formaldehyde solution, embedded in paraffin and cut into sections using a microtome (Leica RM2235). Serial sections (from 2–5 µm thick) from each block were stained to assess spongiform degeneration, brain inflammation, neuronal degeneration, and neuronal loss. The following studies were done:

(a) Brain Vacuolation: The two most affected areas were selected for vacuolation profile and vacuoles were counted in cerebellum and medulla by a method described previously [79].
(b) Brain inflammation: The activated astrocytes were assessed by using a well known astrocytes marker Glial Fibrillary Acidic Protein (GFAP) following a previously described protocol [79]. The activated microglia were stained with a microglial marker IBA-1 as previously used to assess microglial activation in CJD patients [80]. The Digital images were collected on Olympus DP72 Microscope and 3D Histech MIDI (Hungary). The astroglial activation was statistically analyzed by employing a method previously described [3].
(c) Neurodegeneration: The neuronal degeneration and loss was assessed by using Fluoro-Jade C (23062) (AAT Bioquest Sunnyvale Canada) staining, and the NeuN antibody was used for the detection of live neurons via immunohistochemistry analysis. Fluoro-Jade C staining was performed as described by Schmueda et al. [81]. The number of total live neurons was counted as previously described [3].

### 4.10. Transmission Electron Microscopy

The prion infected and control hamster brains were cut into small pieces from different parts of the brain after fixation in 5% glutaraldehyde in 0.1-M sodium cacodylate buffer (pH 7.4) for 4 h at 4 °C. The tissue was rinsed thrice with PBS, and further fixed in 1% OsO4 in 0.1-M sodium cacodylate buffer for 2 h. The Double fixed tissue pieces were dehydrated with a series of ethanol concentrations and acetone and were embedded in resin before polymerization at 60 °C for 48 h. The ultrathin sections were mounted onto copper grids and stained with 4% uranyl acetate and lead citrate. Imaging was performed by using a transmission electron microscope (JEM-1230, Tokyo, Japan) operating at 80 or 120 kV.

### 4.11. Preparation of the Cytoplasmic and Nuclear Extracts

The brain tissue obtained from hamsters was homogenized with ice cold PBS buffer, then the homogenate was centrifuged at 500× *g* for 2–3 min. A cyctoplasmic and nuclear protein extraction kit (AR0106) (Boster Biological Technology, Pleasanton, Canada) was used to set up the cytoplasmic and nuclear extracts according to the previous protocol [3].

### 4.12. Preparation of the Cytoplasmic and Mitochondrial Extracts

Cytoplasmic and mitochondrial extraction was obtained by using NBP2-29448 kit from Novus biological, USA. Briefly, the brain tissue was homogenized by using Dounce-homogenizer and then the tissue suspension was transferred into centrifuge tubes and centrifuged at 3000 rpm for ten minutes at 4 °C. The supernatant was then transferred into another centrifuge tube and the palette was discarded. The supernatant was centrifuged at 12,000 rpm for 30 min at 4 °C. The supernatant (cytosolic fraction) was transferred into a microcentrifuge tube and the pellet was resuspended with 5 mL of ice-cold 1X Suspension Buffer. The suspension was centrifuged at 12,000 rpm for 10 min at 4 °C. The supernatant was discarded and the pellet was resuspended in 5 mL (per gram of original tissue) of Suspension Buffer. Mixed 10 μL of the suspension with 10 μL of staining solution and observed under a microscope. The number of mitochondria was counted using a standard hemocytometer. The mitochondrial suspension was then centrifuged at 12,000 rpm for 10 min at 4 °C and the supernatant was discarded. The mitochondrial pellet was lysed in 1 mL of Complete Mitochondrial Lysis Buffer by mixing end-over-end for 30 min at 4 °C. The mitochondrial extract was centrifuged at 12,000 rpm for 5 min at 4 °C and transferred the supernatant (mitochondrial fraction) into a microcentrifuge tube.

### 4.13. Statistical Analysis

We performed western blot and ELISA on at least 5 animals’ brain homogenates per group. The data obtained are expressed as means ± standard deviation. All the comparisons for the parametric data were carried out by using either one-way ANOVA test, followed by post hoc Tukey’s multiple comparison test, or two-way ANOVA test followed by post hoc Bonferroni multiple comparison test via GraphPad Prism 8 software (Version-8, La Jolla, CA, USA). The in vivo survival study was assessed by Log-rank (Mantel-cox) test using GraphPad Prism 8 software (La Jolla, California, USA) and Image-Pro Plus software (Media Cybernetics, Rockville, MD, USA) was used for immunohistochemistry image analysis to calculate mean optical density (MOD), arbitrary fluorescence units (AFU), and number of cells. *P* < 0.05 was considered statistically significant.

## Supplementary Materials

Supplementary materials can be found at https://www.mdpi.com/1422-0067/20/5/1144/s1. Supplementary Videos S1–S6: Videos showing different stages of prion disease based on severity of signs and symptoms as shown in materials and methodes section. Videos were captured from 79th DPI till 107th DPI, when all the animals in prion-vehicle group reached terminal stage and died. Supplementary Videos S7–S9: Videos showing comparison of the animals in prion infected and non infected groups on 100th day post infection. Supplementary Videos S10–S12: Videos showing novel object finding in prion infected and non infected animals on 100th day post infection. Video S1: Video shows a normal healthy non infected animal. Video S2: Video shows a stage-1 animal with roughcoat on limbs with erect ears. Video S3: Video shows animal in stage-2 with extensive roughcoat on limbs, erect ears and hypersensitive to any noise. Video S4: Video shows animal in stage-3 of the disease with hunchback, circling, and some visible motor abnormalities. Video S5: Video shows animal in stage-4 of the disease with urogenital lesions, paralysis of back legs and imbalanced posture. Video S6: Video shows animal in stage-5 of disease where animal is presented with cachexia and lies in the cage with paddling movement of limbs. Video S7: Video shows prion-vehicle animal (positive control group) on 100th DPI after 3 weeks of appearance of initial signs of the disease. Video S8: Video shows prion-FK506+minocycline animal on 100th DPI after 3 weeks combinatory treatment. Video S9: Video shows no prion-vehicle animal (negative control group) on 100th DPI with no signs of the disease. Video S10: Video shows novel object finding in prion-vehicle animal (positive control group) on 100th DPI after 3 weeks of appearance of initial signs of the disease. Video S11: Video shows novel object finding in prion-FK506+minocycline animal on 100th DPI after 3 weeks combinatory treatment. Video S12: Video shows novel object finding in no prion-vehicle animal (negative control group) on 100th DPI with no signs of the disease.

## Abbreviations

NFAT	Nuclear factor of activated T-cells
IL-1β	Interleukin 1 beta
TNF-α	Tumor necrosis factor alpha
MAPK	Mitogen-activated protein kinase
pCREB	Phosphorylated cAMP response element-binding protein
pBAD	Phosphorylated Bcl2-associated death promoter
NRF2	Nuclear factor–erythroid2-related factor-2
HO-1	Heme oxygenase 1
NF-kB	Nuclear factor kappa-b
TSEs	Transmissible spongiform encephalopathies
vCJD	Variant CJD
PrP^c^	Normal cellular prion protein
PrP^Sc^	Prion protein scrapie
Keap1	Kelch-like erythroid cell-derived protein with CNC homology (ECH)-associated protein 1
CaN	Calcineurin
IL2	Interleukin 2
OF	Open field
NOR	Novel object recognition
SDS-PAGE	Sodium dodecyl sulphate-polyacrylamide gel electrophoresis
ECL	Enhanced chemiluminescence
PBS	Phosphate buffered saline
ANOVA	Analysis of variance
FJ-C	Fluoro-Jade C
DLP1	Dynamin-like protein 1
BAX	bcl-2-like protein 4
Cyt-C	Cytochrome-C
CNS	Central nervous system
IHC	Immunohistochemistry
AD	Alzheimer’s disease
PD	Parkinson’s disease
